# Supplementary material for: MMPs and TIMPs Expression Levels in the Periodontal Ligament during Orthodontic Tooth Movement: A Systematic Review of In Vitro and In Vivo Studies
Source: Int J Mol Sci. 2021 Jun 28;22(13):6967. doi: 10.3390/ijms22136967 (PMC8268288; doi:10.3390/ijms22136967)
Supplement: Supplementary file 1 [file ijms-22-06967-s001.zip › ijms-1257529-supplementary.pdf]

Supplementary Materials

Systematic Review

MMPs and TIMPs expression levels in the periodontal ligament during orthodontic tooth movement: a systematic review of in vitro and in vivo studies

Christian Behm [1, 2], Michael Nemec [1], Fabian Weissinger [2], Marco Aoqi Rausch [1, 2], Oleh Andrukhov [1, *] and Erwin Jonke [1]

[1] Clinical Division of Orthodontics, University Clinic of Dentistry, Sensengasse 2A, 1090 Vienna, christian.behm@meduniwien.ac.at, michael.nemec@meduniwien.ac.at, marco.rausch@meduniwien.ac.at, erwin.jonke@meduniwien.ac.at,
[2] Competence Centre for Periodontal Research, University Clinic of Dentistry, Sensengasse 2A, 1090 Vienna, fabianweissinger95@gmx.at, oleh.andrukhov@meduniwien.ac.at,
* Correspondence: oleh.andrukhov@meduniwien.ac.at

Table S1. Risk of Bias Assessment. Reporting quality of all included *in vitro* studies (adapted from Samuel et al. 2016).

Author	Year	Description of scientific background	Description of objectives	Justification for model	Study design description	Defined experimental outcomes	Ethical statement	Cell maintance condition	Description of measurement precision and variability	Statistical analyse
Behm et al.	2021	✓	✓	✓	✓	✓	✓	✓	X	✓
Bolcato-Bellemin et al.	2000	✓	✓	✓	✓	✓	✓	✓	X	✓
Chen et al.	2013	✓	✓	✓	✓	✓	X	✓	X	✓
Fujihara et al.	2010	✓	✓	X	✓	✓	X	✓	X	✓
Grimm et al.	2020	✓	✓	X	✓	✓	✓	✓	X	✓
Hacopian et al.	2011	✓	✓	✓	✓	✓	✓	✓	X	✓
Huang et al.	2008	✓	✓	✓	✓	✓	✓	✓	X	✓
Jacobs et al.	2014	✓	✓	✓	✓	✓	X	✓	X	✓
Jacobs et al.	2018	✓	✓	✓	X	✓	✓	✓	X	✓
Kook et al.	2011	✓	✓	✓	✓	✓	✓	✓	X	✓
Lisboa et al.	2009	✓	✓	✓	✓	✓	✓	✓	X	✓
Lisboa et al.	2013	✓	✓	X	✓	✓	✓	✓	X	✓
Lisboa et al.	2013	✓	✓	X	✓	✓	✓	✓	X	X
Long et al.	2002	✓	✓	✓	✓	✓	X	✓	X	✓
Ma et al.	2015	✓	✓	✓	✓	✓	✓	✓	X	✓
Narimiya et al.	2017	✓	✓	X	✓	✓	X	✓	X	✓
Nemoto et al.	2010	✓	✓	✓	✓	✓	✓	✓	X	✓
Nettelhoff et al.	2016	✓	✓	✓	✓	✓	✓	✓	X	✓
Proff et al.	2014	✓	✓	✓	✓	✓	✓	✓	X	✓
Redlich et al.	2004	✓	✓	✓	✓	✓	X	✓	X	✓
Saminathan et al.	2012	✓	✓	✓	✓	✓	✓	✓	X	✓
Schröder et al.	2020	✓	✓	✓	✓	✓	✓	✓	X	✓
Tantilertanant et al.	2019	✓	✓	✓	✓	✓	✓	✓	X	✓
Tsuji et al.	2004	✓	✓	✓	✓	✓	✓	✓	X	✓
Wescott et al.	2007	✓	✓	✓	✓	✓	✓	✓	X	✓
Zheng et al.	2012	✓	✓	✓	✓	✓	X	✓	X	✓
Zheng et al.	2019	✓	✓	✓	✓	✓	✓	✓	X	✓
Ziegler et al.	2010	✓	✓	✓	✓	✓	✓	✓	X	✓

✓ Yes (lower bias risk); X No (higher bias risk)

Table S2. Risk of Bias Assessment. Methodological quality of all included *in vitro* studies (adapted from Samuel et al. 2016).

Author	Year	Baseline characteristics similarity / appropriate control group selection	Complete outcome data	No selective outcome reporting	Sample size determination	Statistical analysis	Appropriate/ controlled exposure	Statement of conflict of interest/ funding source	Test system	Test treatment details
Behm et al.	2021	✓	✓	✓	X	✓	✓	✓	✓	✓
Bolcato-Bellemin et al.	2000	✓	✓	✓	X	✓	✓	X	✓	✓
Chen et al.	2013	✓	✓	✓	X	✓	✓	X	✓	✓
Fujihara et al.	2010	✓	✓	✓	X	✓	✓	X	✓	✓
Grimm et al.	2020	✓	✓	✓	X	✓	✓	✓	✓	✓
Hacopian et al.	2011	✓	✓	✓	X	✓	✓	X	✓	✓
Huang et al.	2008	✓	✓	✓	X	✓	✓	X	✓	✓
Jacobs et al.	2014	✓	✓	✓	X	✓	✓	✓	✓	✓
Jacobs et al.	2018	✓	✓	✓	X	✓	✓	✓	✓	✓
Kook et al.	2011	✓	✓	✓	X	✓	✓	✓	✓	✓
Lisboa et al.	2009	✓	✓	✓	X	✓	✓	X	✓	✓
Lisboa et al.	2013	✓	✓	✓	X	✓	✓	✓	✓	✓
Lisboa et al.	2013	✓	✓	✓	X	X	✓	✓	✓	✓
Long et al.	2002	✓	✓	✓	X	✓	✓	X	✓	✓
Ma et al.	2015	✓	✓	✓	X	✓	✓	✓	✓	✓
Narimiya et al.	2017	✓	✓	✓	X	✓	✓	X	✓	✓
Nemoto et al.	2010	✓	✓	✓	X	✓	✓	✓	✓	✓
Nettelhoff et al.	2016	✓	✓	✓	X	✓	✓	✓	✓	✓
Proff et al.	2014	✓	✓	✓	X	✓	✓	✓	✓	✓
Redlich et al.	2004	✓	✓	✓	X	✓	✓	X	✓	✓
Saminathan et al.	2012	✓	✓	✓	X	✓	✓	X	✓	✓
Schröder et al.	2020	✓	✓	✓	X	✓	✓	✓	✓	✓
Tantilertanant et al.	2019	✓	✓	✓	X	✓	✓	✓	✓	✓
Tsuji et al.	2004	✓	✓	✓	X	✓	✓	X	✓	✓
Wescott et al.	2007	✓	✓	✓	X	✓	✓	X	✓	✓
Zheng et al.	2012	✓	✓	✓	X	✓	✓	✓	✓	✓
Zheng et al.	2019	✓	✓	✓	X	✓	✓	✓	✓	✓
Ziegler et al.	2010	✓	✓	✓	X	✓	✓	X	✓	✓

✓ Yes (lower bias risk); X No (higher bias risk)

Table S3: Risk of Bias Assessment of all included *in vivo* studies (adapted from The Joanna Briggs Institute Critical Appraisal Checklist for analytical cross-sectional study)

Authors	Year	Were the criteria for inclusion in the sample clearly defined?	Were the study subjects and the setting described in detail?	Were objective, standard criteria used for measurement of the condition?	Were confounding factors identified?	Were strategies to deal with confounding factors stated?	Were the outcomes measured in a valid and reliable way?	Was appropriate statistical analysis used?
Alikhani et al.	2018	✓	X	✓	✓	✓	✓	✓
Apajalahti et al.	2003	X	X	✓	X	X	✓	✓
Bildt et al.	2009	✓	✓	✓	X	X	✓	✓
Cantarella et al.	2006	✓	X	✓	X	X	✓	✓
Capelli et al.	2011	✓	X	✓	X	X	✓	✓
Grant et al.	2013	✓	X	✓	✓	✓	✓	✓
Ingman et al.	2005	X	X	✓	X	X	✓	X
Ribagin et al.	2012	✓	X	✓	X	X	✓	✓
Rody et al.	2014	✓	X	✓	X	X	✓	✓
Shirozaki et al.	2020	✓	X	✓	✓	X	✓	✓
Surlin et al.	2014	✓	X	✓	X	X	✓	✓
Zhang et al.	2020	✓	X	✓	X	X	✓	✓

✓yes (lower bias risk); X no (higher bias risk
